# Remote Control of Eukaryotic Gene Expression by a Modular Ultrasound‐Responsive RNA Toolkit

**DOI:** 10.1002/anie.202421803

**Published:** 2025-05-19

**Authors:** Fahimeh Charbgoo, Aman Ishaqat, Junlin Chen, Fabian Wiertz, Adrian Kuzmanović, Matthias Bartneck, Fabian Kiessling, Andreas Herrmann

**Affiliations:** ^1^ Institute of Technical and Macromolecular Chemistry RWTH Aachen University Aachen Germany; ^2^ DWI – Leibniz Institute for Interactive Materials Aachen Germany; ^3^ Institute of Pharmacology and Toxicology TU Munich Munich Germany; ^4^ Institute for Experimental Molecular Imaging Uniklinik Aachen, RWTH Aachen University Aachen Germany; ^5^ Department of Medicine III RWTH Aachen University Aachen Germany; ^6^ Center for Biohybrid Medical Systems (CMBS) RWTH Aachen University Aachen Germany

**Keywords:** Gene expression regulation, RNA, Rolling Circle Transcription, Sonogenetics, Ultrasound‐responsive systems

## Abstract

Achieving remote control of biological processes remains a significant challenge in genetics. Although ultrasound has been employed to remotely regulate biological functions by targeting mechanosensitive ion channels, existing systems are constrained by the limited responsiveness of specific channels to specific ultrasound frequencies and their applicability to only a few cell types. Sonogenetics has shown promise for promoter control, thereby regulating gene transcription in eukaryotes. Here, we introduce a new modular toolkit for regulating gene expression using ultrasound‐responsive RNA carriers capable of releasing small molecule modulators in response to a broad spectrum of ultrasound frequencies. The cells contain engineered mRNA structures encoding riboswitches or aptazymes, which respond specifically to these small molecule modulators finally controlling downstream protein expression by biocompatible ultrasound. This toolkit is versatile, functioning across various eukaryotic systems—from yeast to mammalian cells—and offers control over gene expression by regulating mRNA translation. We demonstrated that this sonogenetic toolkit robustly modulates gene expression, achieving up to a six‐fold downregulation of protein levels in response to ultrasound stimulation. By expanding the application of sonogenetics across eukaryotes, this RNA‐based toolkit might provide a promising platform for remotely controlling protein function in specific tissues through on‐demand ultrasound activation in the future.

## Graphical abstract

A modular RNA‐based sonogenetic toolkit enables remote control of gene expression in eukaryotes by releasing small molecule modulators from ultrasound‐responsive carriers. These modulators activate engineered mRNA elements such as riboswitches, leading to tunable protein expression. The system functions across diverse cell types and responds to a wide ultrasound frequency range, offering a versatile platform for biocompatible, non‐invasive regulation of cellular processes.

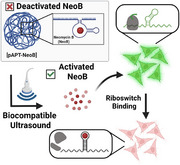

## Introduction

External control of molecular and cellular processes within living organisms is essential for investigating and understanding biology and diseases. The ability to remotely control various cellular functions using external triggers on demand represents a breakthrough in biology and medicine, offering selective diagnosis and therapy, especially for hard‐to‐access organs.^[^ ^1^, ^2^, ^3^, ^4^ ^]^ This field has been advanced through several disciplines, such as optogenetics, magnetogenetics and sonogenetics, which have enabled the manipulation of biomolecular processes.^[^ ^4^, ^5^, ^6^ ^]^ Among these technologies, sonogenetics, which emerged less than a decade ago, exhibits distinctive features and offers unique advantages over the others.^[^ ^7^ ^]^

Sonogenetics facilitates the imaging, manipulation and control of cellular activity through ultrasound (US), by employing genetically encoded US‐responsive elements.^[^ ^4^, ^8^, ^9^, ^10^, ^11^, ^12^ ^]^ US provides precise, cell‐specific and noninvasive spatial and temporal control,^[^ ^13^ ^]^ with high tissue penetration depth, efficacy and safety. It is applicable to a wide range of systems, from cells to mammals and humans. Its frequency and penetration depth can be tuned to different length and time scales,^[^ ^14^ ^]^ allowing for efficient adaptation to specific applications. Furthermore, US has a well‐established history of safety in medical settings, facilitating the translation of sonogenetics into clinical use.^[^ ^14^ ^]^

Recent studies have demonstrated the potential of sonogenetics in programming biological functions by remotely controlling neurons. However, many of its prospective applications remain unexplored.^[^ ^15^ ^]^ Currently, the most commonly used sonogenetic toolkits rely on mechanosensitive ion channels (MSiC).^[^ ^4^, ^14^ ^]^ US can activate these channels to regulate the influx of ions into the cytoplasm, thereby affecting various biological processes such as body movement, gene expression and cancer cell death.^[^ ^7^, ^8^, ^16^ ^]^ Although efforts have successfully enabled the exogenous expression of MSiC in neural and some cancer cells, conferring ultrasound sensitivity to additional cell types both in vitro^[^ ^17^, ^18^, ^19^ ^]^ and in vivo,^[^ ^4^, ^16^ ^]^ there remains a significant need for a modular toolkit to facilitate sonogenetics across a broader range of cells and tissues.

Additionally, each type of MSiC exhibits different levels of US sensitivity and responds to a specific US frequency, including 500 kHz, 2 , 7  and 10 MHz.^[^ ^4^, ^14^ ^]^ Therefore, a modular toolkit that responds to a broad range of US frequencies, ideally using a single US‐responsive element, is needed to overcome the limited spatial resolution caused by the overlap of activation frequencies between exogenously and endogenously expressed MSiCs.^[^ ^4^ ^]^

**Figure 1:**
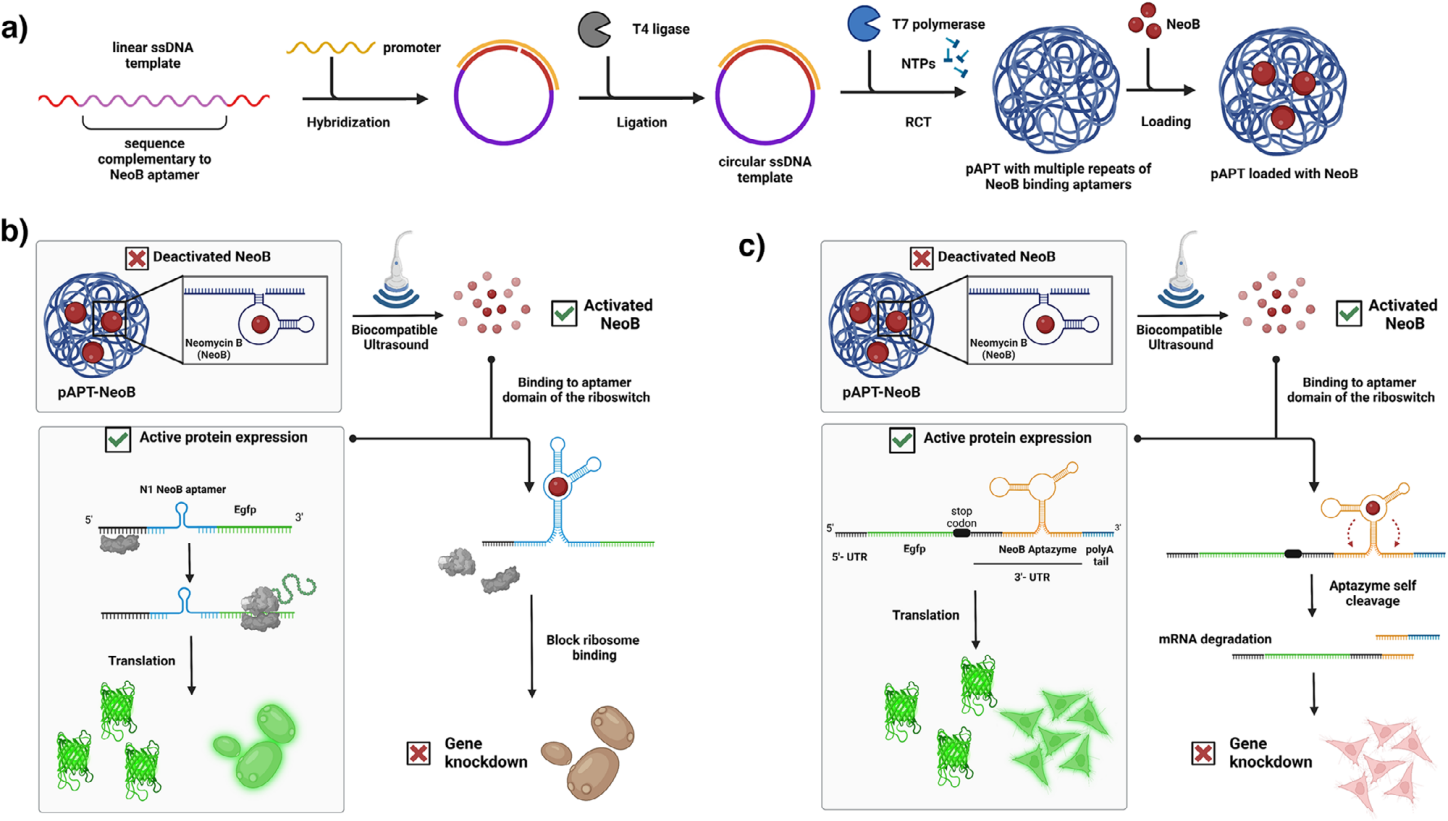
a) Schematic presentation of pAPT production that acts as an ultrasound‐responsive carrier and loading with NeoB cargo. The synthesis starts with a ligation of a single stranded linear DNA template into a circular one with the help of T7 promoter sequence. This is followed by RNA polymerization that leads to pAPT formation which is then be loaded with NeoB cargo by addition of the aminoglycoside. b) Schematic presentation showing the combined use of US with riboswitches to control gene expression and knockdown of EGFP at the translation step in yeast cells. The N1 NeoB aptamer is placed upstream of Egfp. In its unbound state, the ribosome can read the mRNA and translate it into EGFP. When US is used to liberate NeoB from its corresponding pAPT‐NeoB complex, it binds to its aptamer domain in the riboswitch on the mRNA and the bound state cannot be resolved by the ribosome, therefore gene expression is halted. c) Schematic presentation showing the combined use of US with aptazymes to control gene expression and knockdown of EGFP at the translation step in mammalian cells. In the riboswitch, an aptazyme, was incorporated into mRNA to control translation with the small trigger molecule NeoB, which is liberated by US. The riboswitch was comprised of the Neo4‐HHR aptazyme inserted into the 3′‐UTR of Egfp. In the absence of its ligand (i.e., NeoB), the riboswitch is dormant and EGFP is translated. When US is used to activate NeoB from its corresponding pAPT‐NeoB complex, it binds to its aptamer domain in the riboswitch and activates its self‐cleavage leading to mRNA degradation due to dissection of the polyA tail (in blue) leading to EGFP gene downregulation.

Towards this end, we designed and developed such a modular toolkit that can function in a wide variety of eukaryotic cells and responds to a diverse array of US frequencies, thereby enabling unprecedented control over gene expression at the translation level. Our toolkit design leverages the multifunctionality of RNA as both a mechanosensitive carrier material for small molecular mediators and a regulatory element. As the first component, we synthesized high‐molar‐mass polyaptamers (pAPT) comprising repeating units of a RNA nucleic acid aptamer containing the antibiotic neomycin B (NeoB) as illustrated in Figure 1a. In prior work, we demonstrated that the application of US disintegrates both covalent and non‐covalent interactions, including hydrogen bonds and electrostatic interactions, leading to the releases of different types of cargo.^[^ ^9^, ^20^ ^]^ In this work, we utilize the controlled‐release of the small molecule cargo as a regulator of riboswitches genetically engineered in target cells. Our design of US‐responsive riboswitch includes a binding domain for NeoB recognition, either as N1 aptamer in yeast or N4‐HHR aptazyme in mammalian cells. This RNA module can sense the presence of NeoB which can be delivered from its pAPT carrier in a controlled fashion using US. Binding of NeoB leads to gene downregulation at the translation level in yeast (Figure 1b) and mammalian cells (Figure 1c). Our US‐responsive RNA module exhibited robust performance, achieving up to six‐fold gene knockdown. Furthermore, we demonstrated the versatility of our system by showing that it responds precisely to different frequencies of US, including 20 kHz, 1 MHz with low‐intensity focused US (LIFU) and 4–12 MHz with a wideband imaging US device, both in yeast and mammalian cells.

## Results and Discussion

### Generation of an US‐Responsive Carrier

**Figure 2:**
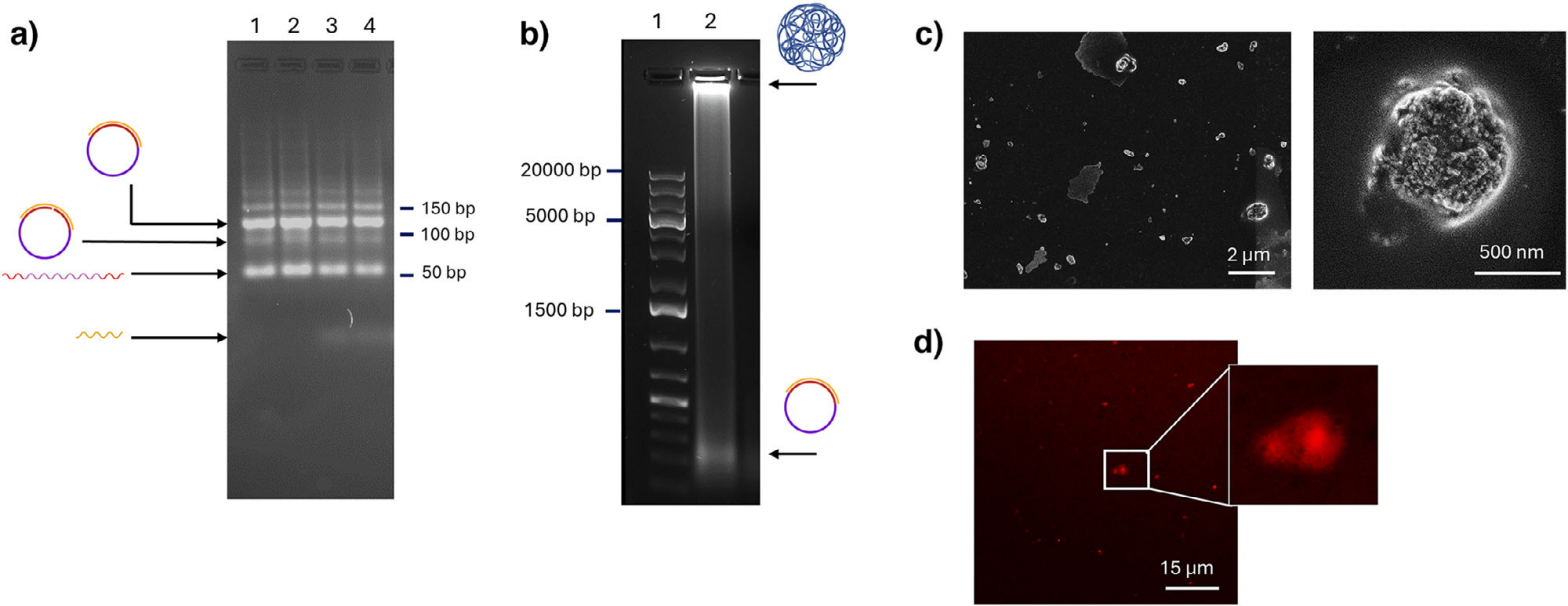
a) Gel electrophoresis (GE) showing the ligation of the linear template into a circular one, which exhibited reduced mobility compared with the linear template. The ultra‐low range gene ruler is used to confirm the size of the bands. Lanes 1 to 4 contain samples from different repeats of the reaction. b) GE showing the production of pAPT via RCT reaction using the circular DNA template. c) Scanning electron microscopy images of pAPT showing its three‐dimensional spherical structure. d) Confocal microscopy image of cy3‐Par‐pAPT showing spherical structures with a red, fluorescent signal confirming the loading of cy3‐Par to the pAPT carrier.

The first aim of our study was to design an US‐responsive carrier, which can be remotely activated by US to release a small bioactive molecule that can penetrate various cell types. For this purpose, we utilized the R23 RNA aptamer (APT) that exhibits high binding affinity and selectivity toward NeoB^[^ ^21^ ^]^ (sequences are listed in Table S1). We employed this aptamer sequence as a repeating unit in the rolling circle transcription (RCT)^[^ ^9^ ^]^ process to produce a high molar mass RNA strand encoding a large number of NeoB binding sites along the backbone. The RCT process was preceded by fabricating a circular single‐stranded DNA (ssDNA) template encoding the APT complementary sequence (Figure 1a) using padlock ligation.^[^ ^22^ ^]^ The formation of the DNA circular template (Figure 2a), as well as the RCT products (Figure 2b) was confirmed using gel electrophoresis (GE) where the resulting RCT products, called polyaptamers (pAPT), exhibited very high molar mass. Moreover, pAPT were observed to have spherical strcutures with the size range of 0.5–1.5 µm, as determined by scanning electron microscopy (SEM) (Figure 2c). Furthermore, we confirmed that pAPT was stable under cell culture conditions and was not degraded by nucleases (Figure S1A).

We proceeded to investigate the impact of ultrasonication on pAPT, utilizing both 20 kHz and 1 MHz US frequencies. GE revealed a decrease in the molar mass of pAPT upon exposure to 20 kHz US, indicating a significant breakage of covalent bonds within the pAPT backbone (Figure S1B). With increasing US exposure time, successive chain scission of pAPT was observed over a period of 30 min. In contrast, 1 MHz LIFU did not result in the breakage of covalent bonds within the pAPT over the same time period (Figure S1C). However, the breaking of non‐covalent bonds was feasible under these sonication conditions. Such intramolecular bonds are the ones to be expected to form between NeoB and its aptamer, and their breakage is the critical step to achieving US‐controlled release of NeoB.

Next, we studied the loading of NeoB cargo by adding it to the synthesized pAPT carrier at different w:w ratios (i.e., NeoB:pAPT at 1:0, 1:0.25, 1:1, 1:2 and 1:6). We determined the loading efficiency by performing a viability test on *Lactococcus lactis (L. lactis)* bacteria that demonstrates the successful inhibition of the bioactivity of NeoB when incorporated into the carrier (Figure S2A). The optimal ratio of NeoB:pAPT was determined to be 1:2 (Figure S2B), which did not result in reduced viability compared to treatment with free NeoB, underlining the successful loading of NeoB to the pAPT carrier. Therefore, the 1:2 ratio of NeoB:pAPT was used for further experiments.

To further confirm NeoB loading into the carrier, we characterized the system using a fluorescently labelled analog of NeoB, that is, cy3‐modified paromomycin (cy3‐Par), which binds to the R23 aptamer with similar affinity. Confocal microscopy revealed spherical red structures with high fluorescent intensity (Figure 2d) exhibiting morphology and size similar to those observed for pAPT in SEM analysis, thereby confirming the successful loading of antibiotics cargo.

Next, we investigated the release of NeoB from the NeoB‐pAPT complex upon exposure to US at 20 kHz and 1 MHz frequencies. To monitor the process, we carried out a viability assay in which the NeoB‐pAPT complex was incubated with *L. lactis* that are resistant to the chosen US conditions alone. After 5 min of 20 KHz ultrasonication, NeoB was released and 57% of bacteria were dead, in a level similar to that upon treatment of cells with pristine NeoB at the same concentration (Figure S3A). Likewise, subjecting bacteria to 30 min of 1 MHz LIFU in the presence of the NeoB‐pAPT complex resulted in 54% dead bacteria in the viability assay (Figure S3B) indicating the successful release of NeoB in its bioactive form. In summary, we demonstrated that the pAPT structure can bind the antibiotic NeoB without leaching and thereby inhibits its bioactivity. Additionally, and in line with our previous findings,^[^ ^9^, ^20^, ^23^ ^]^ polynucleotide scaffolds were found to be US‐responsive. We showed that this pAPT carrier can be triggered by both 20 KHz US and 1 MHz LIFU, enabling the release and activation of NeoB. Hence, we have developed a new US‐responsive polynucleotide carrier system that can be exploited as a toolkit in downstream applications for controlling translation in eukaryotic cells with NeoB as a functional regulatory small molecule mediator, which will be discussed in the following section.

### Functionality of the RNA Toolkit in Yeast

To engineer an US‐responsive RNA riboswitch for use with primary eukaryotic cells, we embedded the well‐established N1 neomycin aptamer^[^ ^24^ ^]^ (N1‐APT) to the 5′‐end of the Kozak sequence of the gene in *Saccharomyces cerevisiae (S. cerevisiae)*. The selection of the N1‐APT as the candidate for an US‐responsive RNA riboswitch in yeast stems from its robust gene regulatory activity (for mammalian riboswitch, the Neo4‐HHR aptazyme was used). Notably, it exhibits a significant conformational change between its pristine and ligand‐bound states.^[^ ^24^ ^]^ This disparity is crucial for its gene regulation capability, as the ligand binding induces large conformational changes that result in substantial thermal stabilization. In contrast, the R23 aptamer is considered to be inactive as a riboswitch due to its pre‐structured free state featuring pre‐formed secondary stem‐loop structure that remains unchanged upon NeoB binding. Hence, R23 aptamer was encoded in the pAPT backbone. Enhanced green fluorescent protein (EGFP) was selected as the reporter gene to monitor the functionality of the RNA toolkit. Although riboswitches have been utilized as ligand‐controlled tools for gene regulation,^[^ ^25^, ^26^ ^]^ they have not been designed for remote control of expression—a capability that can be achieved with our new sonogenetic toolkit.

**Figure 3:**
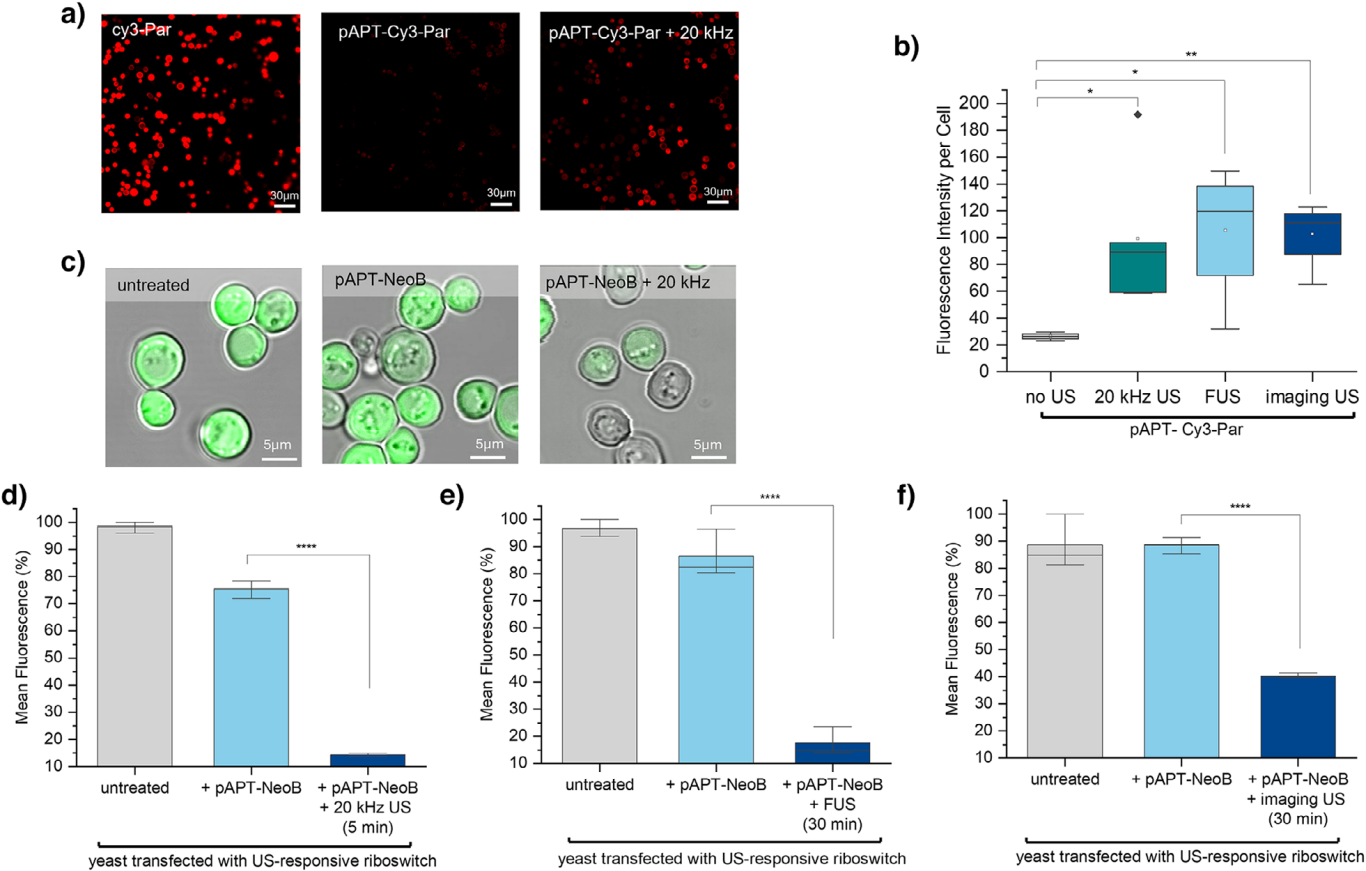
Ultrasound (US)‐controlled gene expression in yeast according to Figure 1b. a) Confocal microscopy images showing yeast cells after incubation with pristine cy3‐Par (left), cy3‐Par‐pAPT (middle) and cy3‐Par‐pAPT plus 20 kHz US exposure for 5 min (right). Cy3‐Par was successfully released from its pAPT carrier after sonication followed by its penetration into the yeast cells. b) Quantification of released cy3‐Par from cy3‐Par‐pAPT into yeast cells upon exposure to different US sources. c) Confocal microscopy images showing US‐induced downregulation of EFGP expression in yeast after ultrasonication with 20 kHz for 5 min. Yeast cells before treatment (left), and after incubation with pAPT‐NeoB both before (middle) and after (right) ultrasonication. Quantification of gene expression by measuring mean fluorescence intensity in yeast after 24‐h incubation with or without ultrasonication with 20 kHz US (d), FUS (e) and imaging US (f). This resulted in six‐fold, two‐fold and two‐fold gene knockdown, respectively.

Initially, we transfected cells with the riboswitch construct along with a plasmid expressing GFP constitutively and treated them with pAPT‐NeoB. In the absence of US, the N1‐APT forms an unstable hairpin structure followed by an unstable helical stem. The small subunit of the ribosome can unfold this structure during the scanning process, leading to active gene translation. Upon exposure to US, the system was switched ON, NeoB was released from pAPT‐NeoB and penetrated the cells. The riboswitch aptamer is stabilized by ligand binding, thus preventing the ribosomal scanning process and resulting in downregulation of gene expression (Figure 1b). The release of cargo followed by cell penetration was confirmed via the fluorescently labeled analog of NeoB liberated from pAPT after being exposed to different US frequencies (Figure 3a,b). We confirmed yeast cell viability after exposure to different concentrations of NeoB (Figure S4A). Furthermore, we confirmed their viability under combined exposure to NeoB and 20 kHz US (Figure S4B). Results demonstrated that up to 80% cells remained viable after treatment with 20 kHz US for the duration of 30 min, in the presence of 200 µM NeoB.

We demonstrated the sonogentic remote control switch mediated by US‐activation of our toolkit (Figure 3c). EGFP expression was knocked down by approximately six‐fold upon exposure to US at 20 kHz frequency (Figure 3d). Furthermore, the use of relevant medical devices such as LIFU (1 MHz) and wideband imaging US (4–12 MHz) led to four‐ and two‐folds of EGFP downregulation, respectively (Figure 3e,f).

To further confirm that gene downregulation is directly related to the riboswitch, which is in turn regulated by NeoB, we assessed gene expression levels in yeast exposed to different concentrations of NeoB and treated with 20 kHz US for different time intervals. One cell population was transfected with EGFP gene plasmid only and lacked the riboswitch (Figure S4C), while the other population was transfected with both EGFP gene plasmid and the riboswitch (Figure S4D). Only the latter cells showed reduction in mean fluorescence at concentrations of NeoB as low as 100 µM and US exposure as short as 5 min. These results demonstrated our toolkit's high specificity and precision since pristine NeoB with US did not affect the expression level of control cells that contain the EGFP reporter gene without the RNA riboswitch element.

### Functionality of the RNA Toolkit for Remote Gene Silencing in Mammalian Cells

After verifying the activity of the US‐responsive RNA module in yeast, we tested our toolkit in human translation systems as a means of remote gene control. We selected the Neo4 aptamer‐ *Schistosoma mansoni* HHR type 3 ribozyme (N4‐HHR aptazyme) and inserted it at the 3′‐end of the stop codon before the polyA signal (sequences are listed in Table S2), specifically 16 nucleotides downstream of the stop codon, of *EGFP* under a constitutively expressed promoter. We chose the 3′‐UTR to maintain a stable secondary structure needed in our riboswitch, as the translation initiation factor 4A (eIF4A) present in the translation initiation complex has gyrase activity, which removes all secondary structures in the 5′‐UTR during the passage of the ribosome while scanning to the start codon in mammalian cells^[^ ^27^ ^]^ (Figure 1c).

The NeoB riboswitch was not used in mammalian cells before due to its low efficiency, which was mainly attributed to the low penetration level of NeoB into the cytoplasm. We demonstrated that by simultaneously applying US and inducing sonoporation, the NeoB penetrated the cells, which allowed the NeoB aptazyme riboswitch to be used in mammalian cells. The red fluorescent signal of the labelled aminoglycoside analogue cargo exhibited a 3.6‐fold increase in the cytoplasm of cancer cells upon incubation with pAPT‐cy3‐Par treated with 20 kHz US (Figure S5).

**Figure 4:**
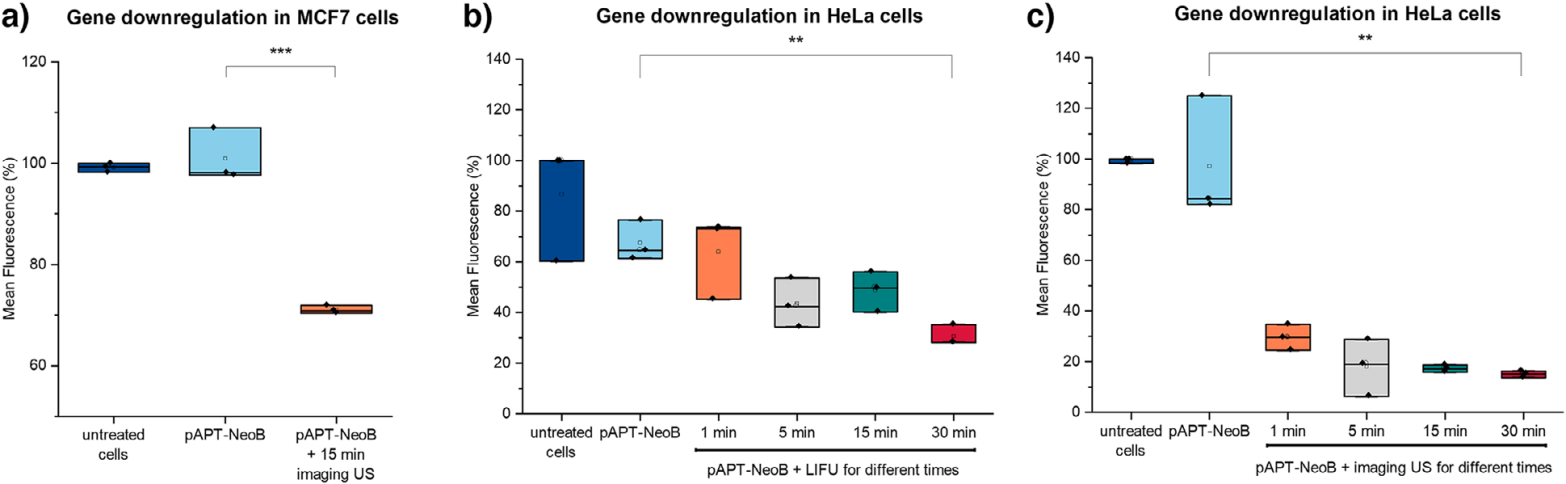
US‐mediated control of protein expression using NeoB as a regulator for the aptazyme in mammalian cells according to Figure 1c. a) EGFP down regulation in MCF7 cells transfected with Neo4‐HHR aptazyme‐EGFP plasmid. Treatment of cells with pAPT‐NeoB plus 15 min of imaging US results in significant decrease in EGFP signal. b) EGFP downregulation in HeLa cells transfected with Neo4‐HHR aptazyme‐EGFP plasmid and treated with pAPT‐NeoB in the absence and presence of LIFU, 1 MHz for different time intervals. c) EGFP downregulation in HeLa cells transfected with Neo4‐HHR aptazyme‐EGFP plasmid and treated with pAPT‐NeoB in the absence and presence of imaging US, 4–12 MHz, for different time intervals.

After confirming the penetration ability of our small molecule regulator, we transformed MCF7 breast cancer cell line with N4‐HHR aptazyme‐EGFP plasmid and treated them with pAPT‐NeoB before exposing them to US at different frequencies and time intervals. Upon US exposure and switching to the ON state, NeoB is released and can bind to the aptamer domain in the riboswitch and activate the HHR self‐cleavage, resulting in the separation of the 3′‐poly‐A tail from the mRNA, leading to its degradation and subsequent gene downregulation (Figure 1c). In the absence of US, NeoB is retained in the pAPT structure, and the Neo4‐HHR aptazyme remains inactive, leading to regular gene expression. Upon cells’ exposure to US, we found a 1.6 and 1.7‐fold downregulation of gene expression using frequencies of 1  and 4–12 MHz, respectively (Figure 4a). In HeLa cells, EGFP expression was downregulated approximately two‐fold after exposure to LIFU at 1 MHz frequency and a focus area of 1 mm (Figure 4b), and 5.8‐fold after exposure to imaging US at 4–12 MHz frequency (Figure 4c). The observed difference in downregulation levels between different US devices can be potentially attributed to the intrinsic focus area of the LIFU (1 MHz) device, compared to non‐focused wideband imaging transducer (4–12 MHz). We further compared the expression level of HeLa cells containing the EGFP plasmid without the aptazyme to cells containing the EGFP plasmid and the aptazyme‐containing riboswitch (Figure S6). In the absence of US, EGFP was expressed in the same manner in both cell types that contain the switch and cells that do not. Furthermore, pristine NeoB and US exposure for cells that lack the riboswitch did not change their EGFP expression. Overall, these findings support that using an RNA‐based US‐responsive element allows achieving remote control over gene expression mediated by small molecules as a regulator, thereby establishing this system as a modular approach to applying sonogenetics to control gene expression in a broad range of eukaryotic cells.

To investigate whether cellular uptake of the RCT product takes place during gene regulation, we conducted fluorescence‐based uptake studies using labelled pAPT in mammalian cells. The results revealed approximately 20% uptake in MCF7 cells and 10% in HeLa cells, with a significantly higher uptake observed at 37 °C compared to 4 °C, indicating the involvement of an active uptake mechanism for a fraction of the construct (Figure S7). However, control experiments in which NeoB was added directly to the culture medium (Figure S4, C and D) demonstrated that gene regulation can also occur without internalization of the RCT product, suggesting that extracellular release and subsequent diffusion of NeoB into cells is sufficient for riboswitch activation. These findings support a dual mechanism involving both extracellular and intracellular NeoB release routes of activation.

## Conclusion

In this study, we developed an US‐responsive sonogenetic RNA toolkit that allows for the downregulation of the target gene in eukaryotic cells upon exposure to a wide range of US frequencies, which offers distinct material properties and release profiles compared to earlier DNA‐based systems and non‐clinically relevant US devices (20 kHz US). Most notably, this study pioneers the use of US‐triggered small‐molecule release to activate a riboswitch‐controlled gene expression system—a novel application that significantly broadens the scope of RNA‐based mechanochemical gene regulation and opens new directions for spatiotemporally precise therapeutic strategies. Our system surpasses the existing MSiC toolkits by expanding the applicability of sonogenetics to different types of eukaryotic cells and regulating gene expression at the translation level. Pan et al. achieved control over gene expression at the transcription level in a sonogenetic manner using the MSiC toolkit by regulating the promotor,^[^ ^16^ ^]^ whereas our RNA toolkit enables direct mRNA regulation, controlling protein translation level. This enables faster response to stimuli and a correspondingly more rapid gene silencing enabled by US. Moreover, our RNA toolkit has proven its function in multiple domains of eukaryotic life, including yeast and mammalian systems, indicating its high potential for numerous applications in biotechnology and biomedicine. Our findings may facilitate various biotechnological applications, such as the production of desired proteins in specific locations in biological systems.^[^ ^28^ ^]^

Our RNA toolkit responds to 20 kHz, 1  and 4–12 MHz US frequencies, enabling the tuning of different parameters of the utilized US device, such as penetration depth, focus and resolution, based on the intended application. Additionally, we anticipate that using different aptamer types with more sophisticated switching activity in human cells will enable a wide range of applications. This work provides a new paradigm for sonogenetics, allowing for mRNA localization and translation control, which may contribute to remotely controlled gene therapy in the future.

## Methods

### Materials

All chemical reagents in this study were of analytical grade and obtained from Sigma, unless specified otherwise. Molecular biotechnology materials, including T4 DNA ligase (5 Weiss U µl^−1^), T7 RNA polymerase (200 U µl^−1^), NTPs (100 mM) and RiboLock RNase inhibitor (40 U µl^−1^), were purchased from Thermo Fisher Scientific, Meerbusch, Germany, unless mentioned otherwise. All oligonucleotide sequences were synthesized and HPLC‐purified by Integrated DNA Technologies (IDT), USA. Neomycin B and paromomycin were bought from Carl Roth, Germany, while Cyanine 3 (Cy3) was obtained from Lumiprobe. The bacterial strain *L. lactis* (ATCC 6538) was obtained from Prof. Bert Poolman, University of Groningen, Netherlands, and the yeast strain *S. cerevisiae* RS453a was obtained from Prof. Beatrix Süß, Technical University of Darmstadt, Germany. HeLa and MCF7 cell lines were obtained from ATCC: The Global Bioresource Center, USA.

### Characterization and Equipment

Optical absorption spectra were measured with a SpectraMax iD3 spectrophotometer and plate reader (SpectraMax M3, Molecular Devices, Germany). Gel images were obtained with an E‐box gel imager (Vilber). Fluorescence imaging was performed using a Leica TCS SP8 confocal microscope (Leica Microsystems, Germany) with an argon laser as excitation source, unless otherwise specified. Fluorescence quantification was performed by flow cytometry using MoFlo Astrios Cell Sorter, Beckman, USA.

### pAPT Preparation

The pAPT template was designed based on RCT as previously described.^[^ ^22^ ^]^ Briefly, the RCT reaction template consisted of three parts, with a middle part complementary to the R23 aptamer sequence and two flanking regions complementary to the T7 promoter sequence. Additionally, spacer nucleotides were added between the middle part and the lateral promoter parts to increase flexibility and enhance the circularization of the template, as well as to provide space between the aptamer units in the pAPT to allow for proper aptamer folding and subsequent efficient binding to its cargo. To prepare the circular template, 100 µM 5′‐phosphorylated linear DNA template (24 µL), 100 µM T7 polymerase promoter (20 µL) and 10X ligation buffer (12 µL;40 mM Tris‐HCl, 10 mM MgCl_2_, 10 mM DTT, 0.5 mM ATP) were added to 60 µL of autoclaved ultrapure water generated using a Milli‐Q Integral Water Purification System (Millipore). The mixture was heated to 95 °C for 5 min and then cooled to room temperature over 30 min. Next, T4 DNA Ligase (4 µL) was added to the reaction mixture at a final volume of 120 µL and incubated at 22 °C for 3 h. The circular DNA was synthesized by padlock ligation. During this process, the T7 promoter strand is hybridized with R23 ligation template at both ends followed by sealing the nick in the circular DNA by T4 DNA ligase. After the ligation, the enzyme was deactivated at 70 °C for 10 min. The sequences used are summarized in Table S1. Agarose GE analysis was performed using 4% agarose gel at 80 V for 90 min. The gels were stained with GelRed Nucleic Acid Gel Stain 10.000X in DMSO (Thermofisher, Germany) to visualize the bands under UV light with gel‐Doc.

The pAPT was synthesized by adding the ligated R23 template (5 µL), 5X transcription buffer (7.5 µL; 40 mM Tris‐HCl, 6 mM MgCl_2_, 10 mM DTT, 10 mM NaCl and 2 mM spermidine), RNase inhibitor (0.5 µL;1 U µL^−1^), 100 mM NTPs (5 µL) and RNAse/DNAse free water (9 µL). The solution was mixed by pipetting, then T7 polymerase (3 µL; 5 U µL^−1^) was added and incubated at 37 °C for 9 h, followed by enzyme deactivation at 70 °C for 10 min. The fabrication of pAPT was verified by GE using 1% agarose gel after running for 60 min at 120 V. The concentration of pAPT was measured at 280 nm by Nanodrop, Thermofisher, Germany. Moreover, the stability of the pAPT and its resistance to nuclease degradation were studied at different time intervals during the growth of *S. cerevisiae* and mammalian cells by treating the pAPT with GM17 and DMEM with 10% foetal calf serum and incubation at 30 °C and 37 °C with 5% CO_2_, respectively. The pAPT in ultrapure water was used as the control of the experiment.

### Scanning Electron Microscopy (SEM)

To study the morphology of the pAPT, a droplet of the reaction solution was applied to a carbon coated copper grid. After drying, a layer of carbon with 3 nm thickness was deposited on the samples using a high vacuum sputter coater (Leica EM ACE600). The samples were then imaged using a HRSEM Hitachi SU9000. The acceleration voltage was set to 2 kV and the emission current was 5 µA.

### Loading of NeoB to pAPT

To determine the optimal w/w ratio of NeoB:pAPT for complete loading of all the NeoB quantity, five different NeoB:pAPT ratios were tested. We started with varying volumes of 2000 ng µl^−1^ pAPT (0; 2; 8; 32; 64 and 200 µL) and completed the volume as needed to reach a final volume of 200 µL using 10 mM sodium phosphate buffer (pH 6.8). These samples were heated to 95 °C for 5 min and maintained at 80 °C for 30 min to prevent rapid refolding. Subsequently, 2 µL of 50 mM NeoB solution in 10 mM sodium phosphate buffer (pH 7.4) were added, and the mixture was allowed to cool to room temperature over 60 min with gentle homogenization by pipetting every 15 min. The NeoB:pAPT weight per weight ratios were 1:0, 1:0.06, 1:0.25, 1:1, 1:2 and 1:6, respectively. To investigate the formation of NeoB‐pAPT, a viability test on *L. lactis* was performed. The bacteria were cultured in M17 media (comprising Pancreatic Digest of Casein, Soy Peptone, Beef Extract, Yeast Extract, ascorbic acid, magnesium sulphate, disodium‐β‐glycerophosphate) supplemented with 1% of glucose (GM17) at 30 °C. The overnight bacterial culture was diluted in 2X GM17 media to achieve an optical density (OD) of 0.4. In a 96‐well plate, 200 µL of the diluted bacterial suspension and 200 µl of NeoB‐pAPT complexes with different w/w ratios were added and incubated overnight at 30 °C. Then, the OD of the cells was measured by a plate reader at 600 nm. Untreated cells and cells treated with the same concentration of pristine NeoB were used as negative and positive controls, respectively. Based on the results, a NeoB:pAPT w/w ratio of 1:2 was selected for all further experiments as it consistently achieved robust NeoB loading without leaching of the antibiotic. Confocal microscopy was used to further study NeoB loading using cyanine 3‐fluorophore‐labeled paromomycin (cy3‐Par) as an analogue of NeoB that binds to the R23 aptamer with similar affinity. The cy3‐Par‐pAPT complex was prepared using the aforementioned protocol and the samples were diluted at a ratio of 1:500 in ultrapure water. Twenty microliter of the cy3‐Par‐pAPT were placed on a glass slide, covered with a micro cover slip and imaged using confocal microscopy with a 514 nm argon laser for excitation.

### Ultrasonication Experiments

Ultrasonication experiments were performed using different devices. For 20 kHz US, a Qsonica Q125 sonicator equipped with a 3‐mm‐diameter microtip probe (A12628PRB20) was used to sonicate samples in 1 mL ultrasonication tubes at 50% amplitude. The input sonication energy *E* (in the unit J) was recorded during sonication and used to calculate the sonication power *P *= *E t* ^−1^ and power intensity *IP* = *P A* ^−1^, where *t* denotes the effective sonication “on” time, and *A* is the area of the probe tip. Pulsed sonication (1.0 s on, 1.0 s off) was used. Over the full duration of the sonication, this corresponds to *t* = 1800 s, *E* = 4551 J, *P* = 2.53 W, *A* = 0.07 cm^2^ and *IP* = 36.14 W^.^cm^−2^. The vessel was placed in an ice bath to maintain the temperature inside the tubes at 6°C–9 °C throughout sonication.

For the other US experiments with biocompatible US, we used two devices operating at 1  and 4–12 MHz. To ensure their biocompatibility, we employed parameters within established safety guidelines. We previously validated their safety in vitro^[^ ^20^, ^29^ ^]^ and in vivo,^[^ ^20^ ^]^ with no adverse effects on cell viability or tissue health under the applied conditions.

Sonication experiments at 1 MHz were carried out using a low‐intensity focused ultrasound (LIFU) system, which comprised a waveform generator with an integrated oscilloscope function (Model SDS1202X‐E, Siglent. EU, Helmond, Netherlands), a second waveform generator (Model 33622A, Keysight Technologies, Böblingen, Germany), a radiofrequency (RF) broadband power amplifier (Model AG1021, T&C Power Conversion, Rochester, New York, USA) and a waterproof 1 MHz focused immersion transducer (Model V308‐SU, Olympus Europa SE & Co. KG, Hamburg, Germany) in a water tank. The transducer's centre had a distance of 2.5 cm from the ultrasonic transparent 24‐well plate (SARSTEDT AG & Co. KG, Nümbrecht, Germany), allowing the focus point to be aligned to the height of the samples. The acoustic parameters involved frequency (1 MHz), pulse repetition frequency (2 kHz), sonication time (varying between 1 and 30 min) and peak rarefaction pressure (914.8 kPa for the mammalian cells and 6.57 MPa for *S. cerevisiae*) at focus point. In addition to the peak rarefaction pressure, the ultrasound intensity was described by the mechanical index (MI = 0.91 and 6.57, for mammalian cells and yeast, respectively).

Sonication experiments at 4–12 MHz were performed in 60 mm Petri dishes using the Lumify Ultrasound System with an L12‐4 linear array transducer (Philips, Netherlands) with a frequency range of 4–12 MHz using the musculoskeletal (MSK) mode with its default predefined settings. Sonication was performed in cell culture media or sodium phosphate buffer solution in which the Lumify front surface (i.e., acoustic window) was immersed. The distance between the acoustic window and the sample was adjusted to 1.5 cm for the cells in the Petri‐dishes.

### Effects of US on pAPT and NeoB‐pAPT

To investigate the effects of ultrasonication on pAPT, GE was performed with pAPT before and after exposure to either 20 kHz or 1 MHz US at the indicated time intervals using 1% agarose gel in 1X TAE buffer. To study if US mediates the release of NeoB from the NeoB‐pAPT complex, we performed a viability assay on *L. lactis* as described above, before and after exposure of either 20 kHz US or 1 MHz US for the indicated time intervals. Untreated cells, cells treated with NeoB and cells treated with pAPT were used as controls for the experiment.

### Assembly of DNA Plasmid Constructs

For the yeast experiments, N1 neomycin aptamer sequences were cloned into a pWHE601 plasmid (received from Prof. Süß, TU‐Darmstadt, Germany). The N1 sequence was inserted by cutting the pWHE601 plasmid with AFlII and MscI restriction enzymes at the 5′‐ and 3′‐ends, respectively. In the mammalian cell experiments, the Neo4 aptamer‐Schistosoma mansoni HHR type 3 ribozyme (Neo4‐HHR aptazyme) was inserted into the pHA‐EGFP‐HaloTag2 plasmid (Addgene, Plasmid #41 742) by cutting with SgrAI and NotI restriction enzymes. The Neo4‐HHR aptazyme constructs were designed to separate the polyA signal from the gene after US exposure and activation of NeoB. All constructs were amplified by cloning in *E. coli* DH5α. Plasmids or DNA fragments were purified using Sigma Miniprep or GE Healthcare (cytivalife) extraction purification kits. All insertions were either ordered as a gBlock gene fragment or amplified from laboratory plasmids through PCR. Before and after experimentation, all vectors were sequenced using Sanger sequencing performed by Microsynth seqlab, Göttingen, Germany.

### Yeast Cell Culture Growth

Unless otherwise specified, liquid yeast cultures were grown either on 24‐well plates or in falcon tubes, under continuous shaking conditions at 200 rpm at 30 °C, in either YPD or synthetic complete (SC)‐dropout medium supplemented with yeast nitrogen base without amino acids but with ammonium sulphate and glucose (50% w/v). The SC‐dropout media contained nine amino acids, with all standard amino acids present at 76 mg L^−1^, except for leucine which was present at 380 mg L^−1^, and additionally included adenine (18 mg L^−1^), inositol (76 mg L^−1^) and p‐aminobenzoic acid (8 mg L^−1^).

### Effects of NeoB and US on Yeast Viability

To investigate the effect of NeoB on yeast viability, cells were grown overnight, diluted to OD600 = 0.2, and treated with varying concentrations of NeoB, (0, 10, 20, 100, 200, 500 and 1000 µM) in triplicates. After overnight incubation, cell viability was assessed by measuring OD using a plate reader. To assess the simultaneous effects of NeoB and US on yeast cell viability, cells were grown over night, diluted to OD600 = 0.2 at a volume of 1 mL for each sample and transferred into microtubes. The cells were then treated with NeoB at 0, 100 and 250 µM, with each sample prepared in triplicate. Two of the triplicates were irradiated with 20 kHz US for 15 and 30 min, respectively. After overnight incubation, OD600 of the cells was measured.

### Yeast Transformation and Experimentation

Yeast transformations were carried out using the Frozen‐EZ Yeast Transformation II Kit (Zymoresearch, Germany). The resulting strains carried either the pWHE601 or the pWHE60‐N1 aptamers, and were named as positive control cells and US‐responsive RNA riboswitch‐GFP cells (US‐RNA riboswitch‐GFP), respectively. After transformation, the cells were plated on SC‐dropout medium, which was auxotrophic for uracil supplement since the pWHE601 has the URA3 gene to restore it. URA3 encodes orotidine 5′‐phosphate decarboxylase (ODCase), an enzyme that catalyzes one reaction in the pyrimidine ribonucleotides biosynthesis pathway. To investigate the effect of NeoB concentration and the period of US exposure on gene expression levels, the positive control cells, and the US‐RNA riboswitch‐GFP cells were grown overnight and diluted to OD600 = 0.2 in a volume of 1 mL for each sample and transferred into microtubes. The cells were then treated with 0, 100 and 250 µM NeoB. Each sample was prepared in triplicate, two of which were irradiated with 20 kHz US for 15 and 30 min, respectively. Afterwards, the cells were incubated for 48 h and the fluorescent signal of EGFP was measured by flow cytometry. Untreated cells were used as negative control.

### Release of NeoB and Cell Penetration in Yeast

To study the release of NeoB from NeoB‐pAPT complex and its subsequent penetration into cells, two mixtures were prepared by adding 69 µL of cy3‐Par‐pAPT, 50 µL of wild‐type RS435α cells with OD600 = 2.2, 60 µL of 10X YPD and 421 µL sterile water. One mixture was incubated immediately and the other was incubated after 5 min of 20 kHz US exposure. Cy3‐Par‐pAPT was synthesized with 10 µM cy3‐Par to avoid too intense fluorescent signal. The tested conditions included untreated cells, cells treated with 10 µM cy3‐Par, cells treated with 10 µM NeoB, cells treated with 10 µM NeoB‐pAPT and cells treated with 10 µM NeoB‐pAPT and 5 min of 20 kHz US exposure. After 24 h, samples were visualized using confocal microscopy, and fluorescent signal quantification was performed using image J, version 1.53. The percentage of cells showing fluorescence was determined from at least five images.

### Functionality of the RNA Toolkit in Yeast

The positive control and US‐RNA riboswitch‐EGFP cells were treated with NeoB and NeoB‐pAPT RNA complex with and without US exposure at frequencies of 20 kHz, 1  and 4–12 MHz. The cells to be exposed to 20 kHz US were cultured in tubes, the cells to be exposed to 1 MHz US were cultured on 24‐well ultrasonic transparent plates and the cells to be exposed to 4–12 MHz were cultured in bacterial grade Petri dishes. The samples included RS435α cells as negative control, the positive control cells, positive control cells with 100 µM NeoB plus 20 kHz US for 5 min, US‐RNA riboswitch‐GFP cells with 100 µM NeoB, US‐RNA riboswitch‐GFP cells with 100 µM NeoB‐pAPT, US‐RNA riboswitch‐GFP cells with 100 µM NeoB‐pAPT plus 20 kHz US for 5 min, US‐RNA riboswitch‐GFP cells with 100 µM NeoB‐pAPT plus 1 MHz US for 30 min and US‐RNA riboswitch‐GFP cells with 100 µM NeoB‐pAPT plus 4–12 MHz US for 30 min. After 48 h incubation, samples were analysed using flow cytometry, and the samples exposed to 20 kHz US were also visualized using confocal microscopy. Timetable studies of different US frequencies on US‐RNA riboswitch‐GFP cells were performed, and the best outcomes for each frequency were considered for all further experiments. Fluorescence normalization was performed using the following calculation: Fluorescence of treated US‐RNA‐GFP cell/fluorescence of the untreated US‐RNA‐GFP cells × 100.

### Release of NeoB and Cell Penetration in Mammalian Cells

To test the penetration of NeoB into MCF7 breast cancer cells after US exposure, cells were cultured and treated with either cy3‐Par, cy3‐Par‐pAPT or cy3‐Par‐pAPT plus 20 kHz US for 5 min. The cells were subsequently incubated in DMEM supplemented with 10% FCS for 24 h followed by confocal microscopy imaging. Untreated cells were used as negative control. Three to five representative fields per condition were imaged, yielding quantifiable data from approximately 150–200 cells per group. Next, the effect of 1 mM NeoB and 1 MHz US exposure on gene expression levels was investigated by transfecting the pHA‐Halotag‐EGFP plasmid into the cells using Lipofectamine 2000 transfection reagent, as per the manufacturers' instructions. After 4 h of incubation, the medium was changed to DMEM supplemented with 10% FCS and 1 mM NeoB, followed by cells’ exposure to 1 MHz US. After 48 h of incubation, cells were detached using TrypLE Express and resuspended in 2% FCS in PBS for flow cytometric analysis.

### Functionality of the RNA Toolkit in Mammalian Cells

To study the effect of 1  and 4–12 MHz US on gene regulation in mammalian cells, 70% confluent MCF7 and HeLa cells were transfected with the HA‐Halotag‐EGFP plasmid containing the Neo4‐HHR aptazyme (pHalo‐EGFP‐N‐HHR) using Lipofectamine 2000 transfection reagent in 24‐well plates and 65 mm petri dishes, respectively. For the experiments with 1 MHz US, 1 × 10^5^ cells were seeded in 24‐well US transparent plates. After 24 h of incubation at 37 °C, 500 ng (measured by NanoDrop UV‐Vis spectrophotometer) of the pre‐treated HA‐Halotag‐EGFP and pHalo‐EGFP‐N‐HHR in lipofectamine 2000 were added to the cells and further incubated in serum free opti‐MEM media for 4 h at 37 °C. Then, the medium was changed to either DMEM with 10% FCS and 1 mM NeoB, DMEM with 10% FCS and NeoB‐pAPT or DMEM with 10% FCS and 100 g mL^−1^ hygromycin. The latter was used for cells which were transformed with the plasmids but not treated with either NeoB or NeoB‐pAPT. After that, the cells were exposed to 1 MHz US for different time intervals including 0, 1, 5, 15 and 30 min. Untreated cells, cells transformed with HA‐Halotag‐EGFP, and cells transformed with pHalo‐EGFP‐N‐HHR without US exposure served as controls. After 48 h of incubation at 37 °C, the cells were detached and analysed by flow cytometry as previously described. For the experiment with 4–12 MHz US, cells were seeded on 65 mm Petri‐dishes with a primary seeding number of 8 × 10^5^ and irradiated with a wideband US imaging device (i.e., Lumify). Cells without US exposure were cultured in standard 24‐well plates.

### Cellular Uptake by Mammalian Cells

To study the in vitro uptake of the pAPT by cells, MCF7 (ATCC HTB‐22) and HeLa cells (ATCC CCL‐2) were used. The cells were cultured in Dulbecco's modified Eagle medium (DMEM, #P04‐03550, PAN Biotech, Aidenbach, Germany), supplemented with 10% FCS (#P30‐3033, PAN Biotech, Aidenbach, Germany), 100 U mL^−1^ penicillin, and 100 µg mL^−1^ streptomycin (#P06‐07100, PAN Biotech, Aidenbach, Germany), at 37 °C, 5% CO_2_ and 95% relative humidity. Upon reaching full confluency, cells were incubated with AF488‐pAPT at a final concentration of 4.5 µg mL^−1^. After 4 h of incubation at either 4 °C or 37 °C, cells were collected using trypsin/EDTA (#P10‐023100, PAN Biotech, Aidenbach, Germany). Cells were washed once in Dulbecco's Phosphate Buffered Saline (DPBS, #P04‐36500, PAN Biotech, Aidenbach, Germany) to remove excess pAPT and stored in DPBS until flow cytometric analysis.

Flow cytometric analysis was performed using a BD FACSCanto II flow cytometer (BD Biosciences, Franklin Lakes, USA). One crore events were recorded for each sample with forward scattering light and sideward scattering light set to linear scale, and voltages set at 319  and 527 V, respectively. The AF488 fluorescent signal was detected using a logarithmic scale, with the 525/50 detection filter set to 440 V. The data analysis was conducted using FlowJo 10.9.0 (BD Biosciences, Franklin Lakes, USA).

## Author Contributions

F.C. conceived the project, designed and performed experiments, analysed data, and wrote the manuscript draft. J.L. performed experiments with LIFU and F.K. supervised them. F.W. performed the stability experiments and analysed the data. F.W. prepared the materials for the cellular uptake experiments, AK performed the cellular uptake experiments in mammalian cells and analysed the data. A.I. analysed the data, visualized them and wrote the manuscript. M.B, A.I and A.H. reviewed and revised the manuscript. A.H. supervised the research project.

## Conflict of Interests

The authors declare no conflict of interest.

## Supporting information



Supporting Information

## Data Availability

The data that support the findings of this study are available from the corresponding author upon reasonable request.
