# Supplementary material for: Remote Control of Eukaryotic Gene Expression by a Modular Ultrasound‐Responsive RNA Toolkit
Source: Angew Chem Int Ed Engl. 2025 May 19;64(29):e202421803. doi: 10.1002/anie.202421803 (PMC12258680; doi:10.1002/anie.202421803)
Supplement: Supplementary file 1 — Supporting Information [file ANIE-64-e202421803-s001.docx]

**Supporting Information**

Remote Control of Eukaryotic Gene Expression by a Modular Ultrasound-Responsive RNA Toolkit

Fahimeh Charbgoo, Aman Ishaqat, Junlin Chen, Fabian Wiertz, Adrian Kuzmanović, Matthias Bartneck, Fabian Kiessling, Andreas Herrmann


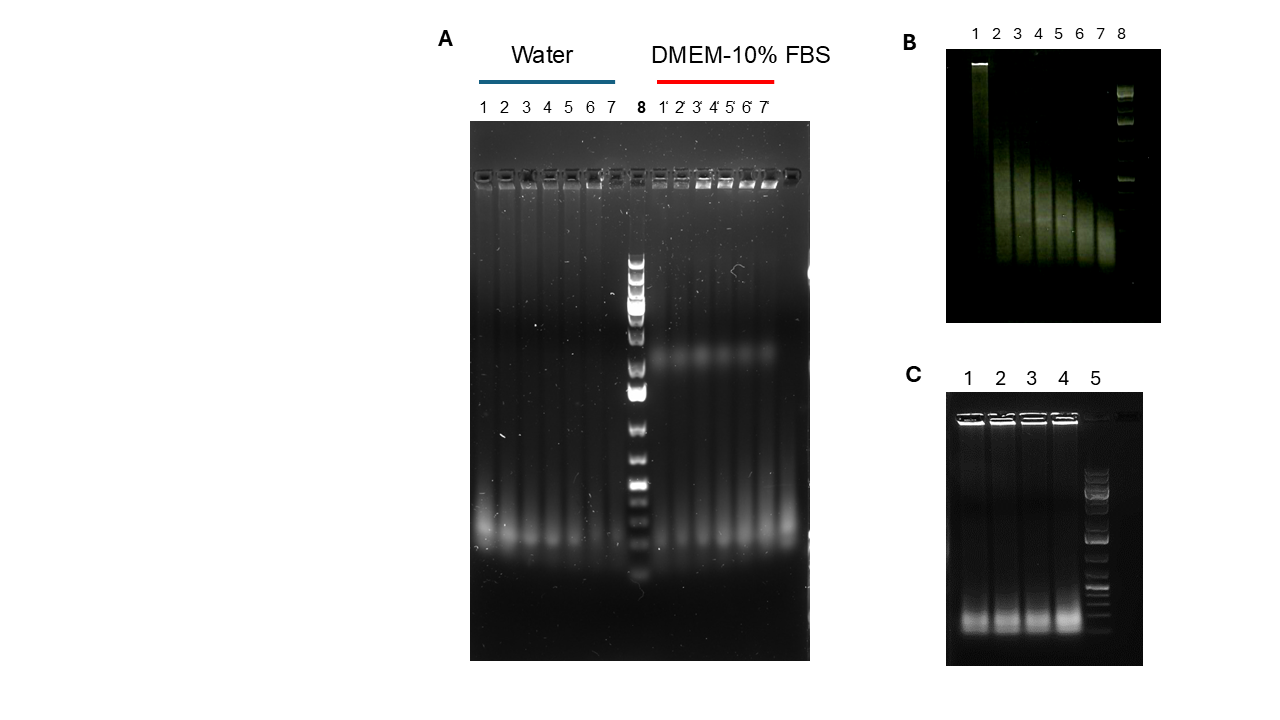


Figure S1.

(**A**) GE demonstrating stability of pAPT against nuclease activity when incubated in DMEM 10% FBS media (at 37 °C, 5% CO_2_) for a total duration of 48 h. Lanes 1 to 6 and lanes 1’ to 6’ are samples taken at 48, 24, 8, 4, 2 and 0 h, respectively; lanes 7 and 7’ is after sonication with 20 kHz US for 30 min; lane 8 is kb plus DNA ladder. (**B**) Gel electrophoresis showing pAPT-NeoB after 20 kHz US exposure for different times. Lane1: pAPT-NeoB; lanes 2 to 7 pAPT-NeoB after 20 kHz US exposure for 1, 2, 4, 8, 15 and 30 min, respectively; lane 8: GeneRuler 1 kb DNA Ladder. (**C**) Gel electrophoresis showing pAPT-NeoB after LIFU exposure for different times. Lane1: pAPT-NeoB; lanes 2 to 4 pAPT-NeoB after LIFU exposure for 5, 15 and 30 min, respectively; lane 5: GeneRuler 1 kb DNA Ladder.


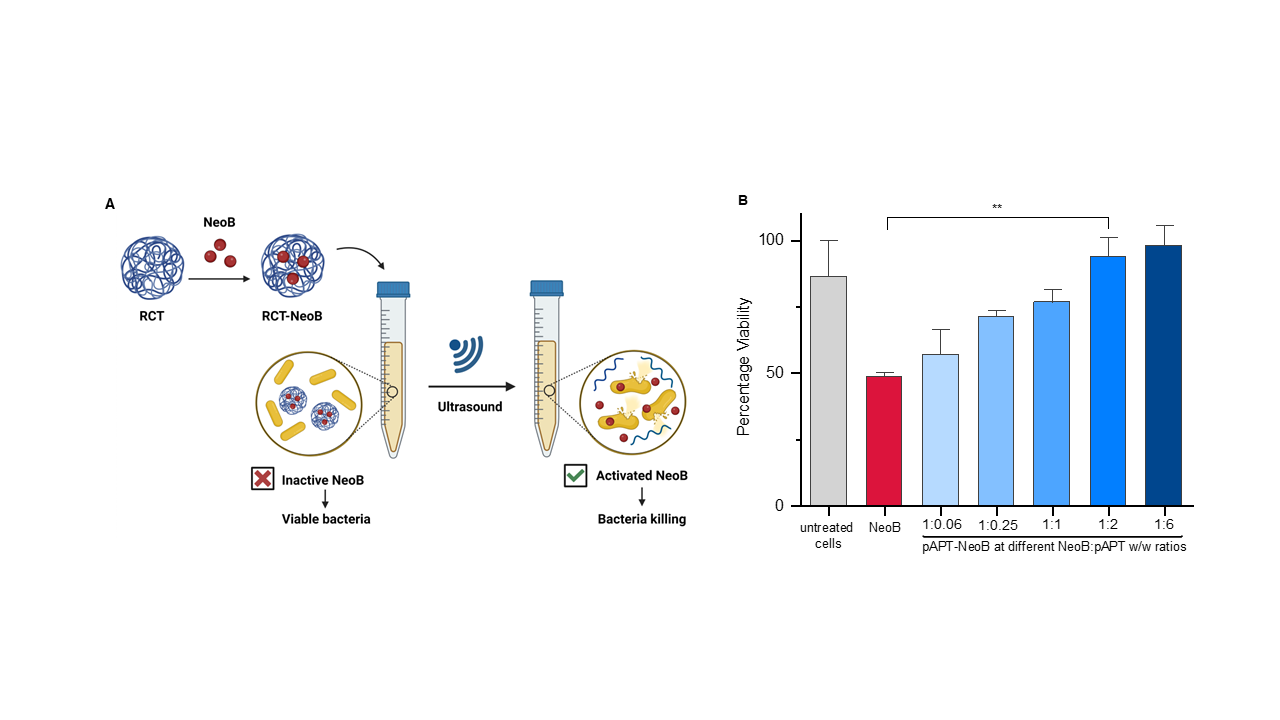


Figure S2.

Loading and release of NeoB on pAPT carrier. (A) Schematic presentation of NeoB loading onto pAPT carrier resulting in pAPT-NeoB complex that binds and inactivates NeoB. Ultrasonication results in NeoB release in its bioactive form leading to bacteria killing. (B) Viability assay in *L. lactis* using different weight/weight ratios of NeoB:pAPT, with NeoB final concentration of 200 µM.


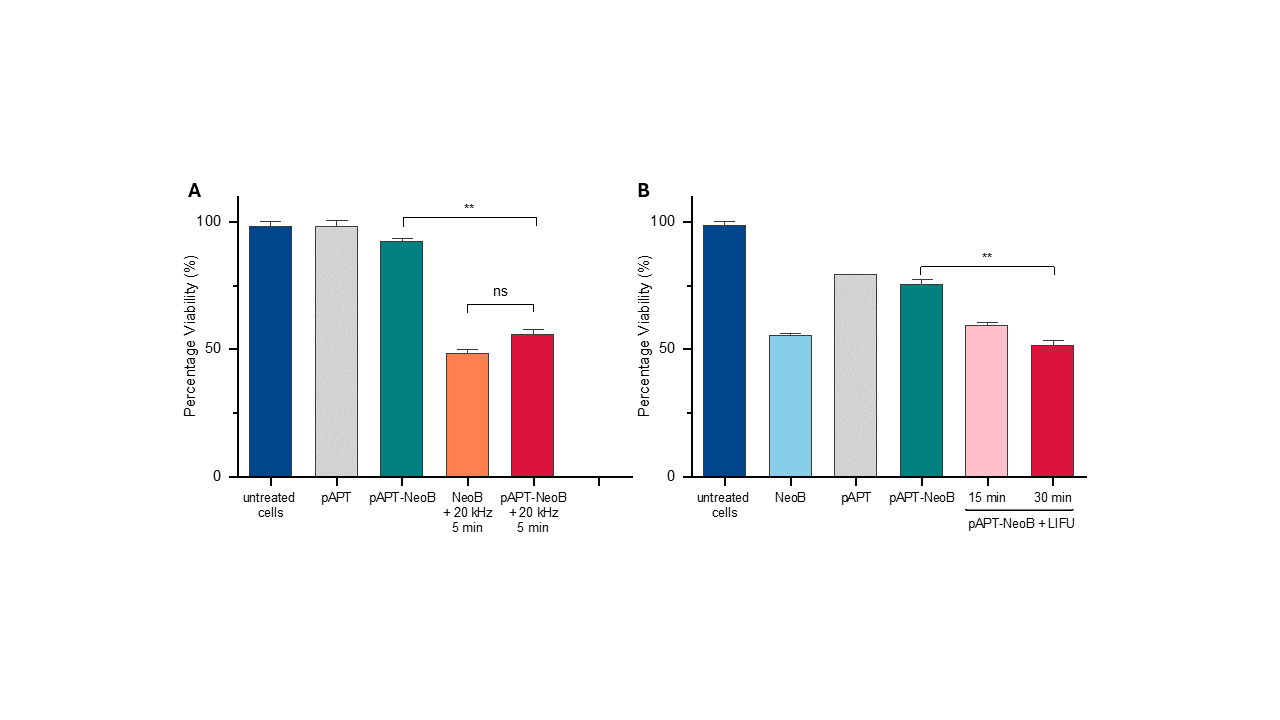


Figure S3.

Release of NeoB from pAPT-NeoB complex prepared at w/w ratio of 1:2 (NeoB:pAPT). (**A**) NeoB release from pAPT-NeoB complex after 20 kHz US exposure shown by viability assay in *L. lactis* under different conditions. (**B**) NeoB release from pAPT-NeoB complex after LIFU exposure shown by viability assay in *L. lactis* under different conditions.


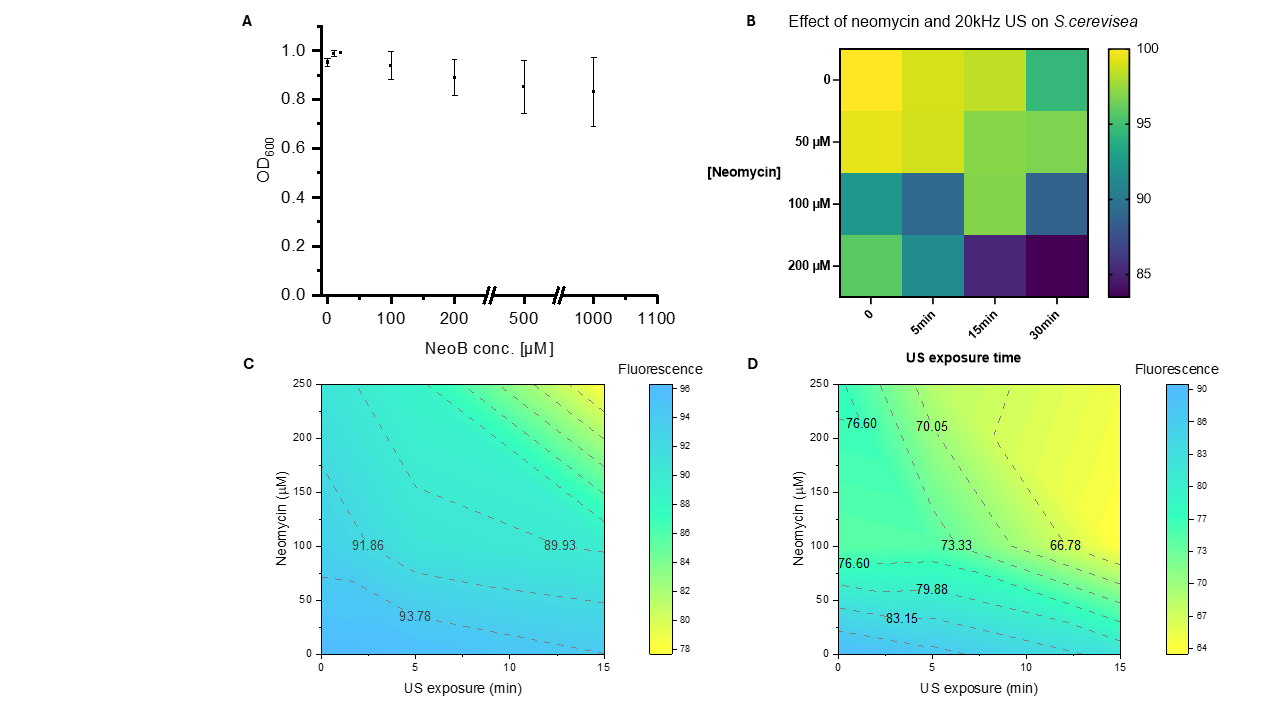


Figure S4.

(**A**) The effect of NeoB at different concentrations on the cell viability of *S. Cerevisiae*. (**B**) The simultaneous effect of NeoB at different concentrations with 20 kHz US exposure at different time intervals on the cell viability of *S. Cerevisiae.* (**C**) The simultaneous effect of NeoB at different concentrations with 20 kHz US exposure at different time intervals on gene expression in yeast cells expressing pWHE601 named as control positive cells. (**D**) The simultaneous effect of NeoB at different concentrations with 20 kHz US exposure at different time intervals on gene expression in yeast cells expressing EGFP and US-responsive riboswitch.


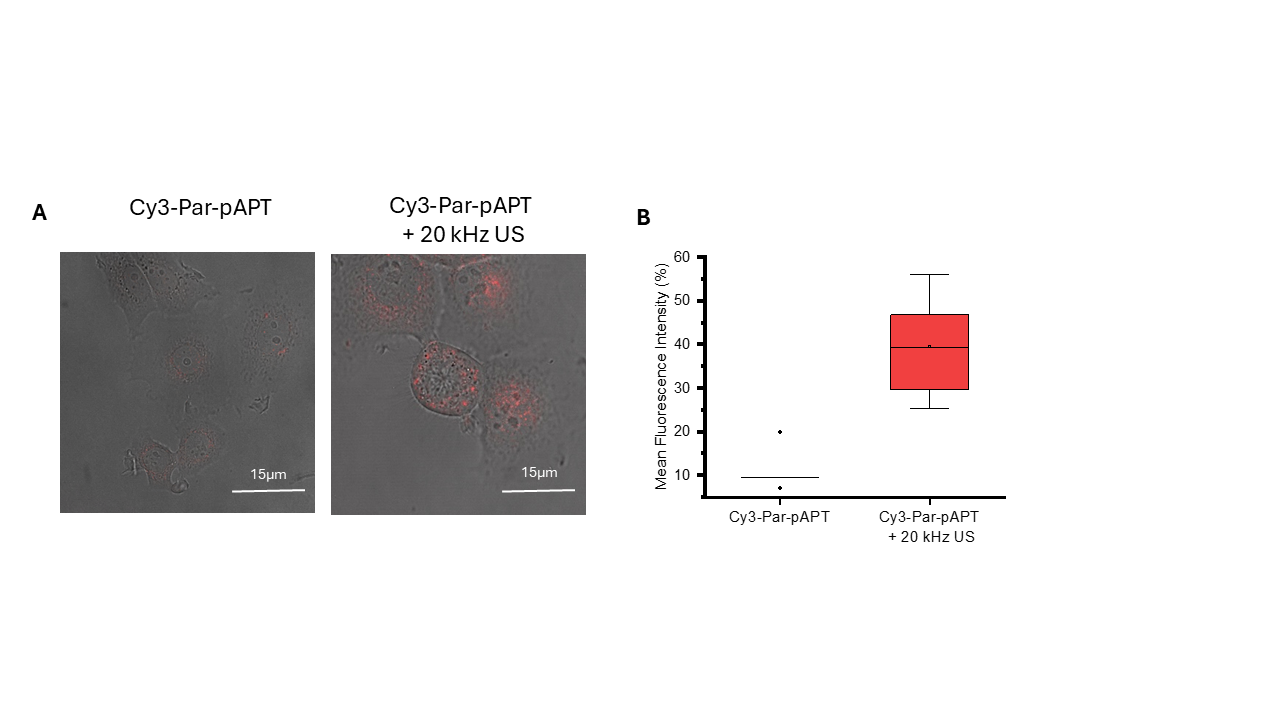


Figure S5.

Cargo release and penetration into mammalian cells after ultrasonication. (**A**) Confocal microscopy images showing MCF7 breast cancer cells incubated either with cy3-Par-pAPT (left) or cy3-Par-pAPT treated with 20 kHz US for 5 min (right). In the latter, red signal inside the cytoplasm demonstrates the penetration of the released cy3-Par across the plasma membrane. (**B**) Quantification of the mean fluorescence of cy3-Par in the confocal microscopy images of cells treated with cy3‑Par‑pAPT with and without the exposure to 20 kHz US for 5 min, following 24 h incubation with cells.


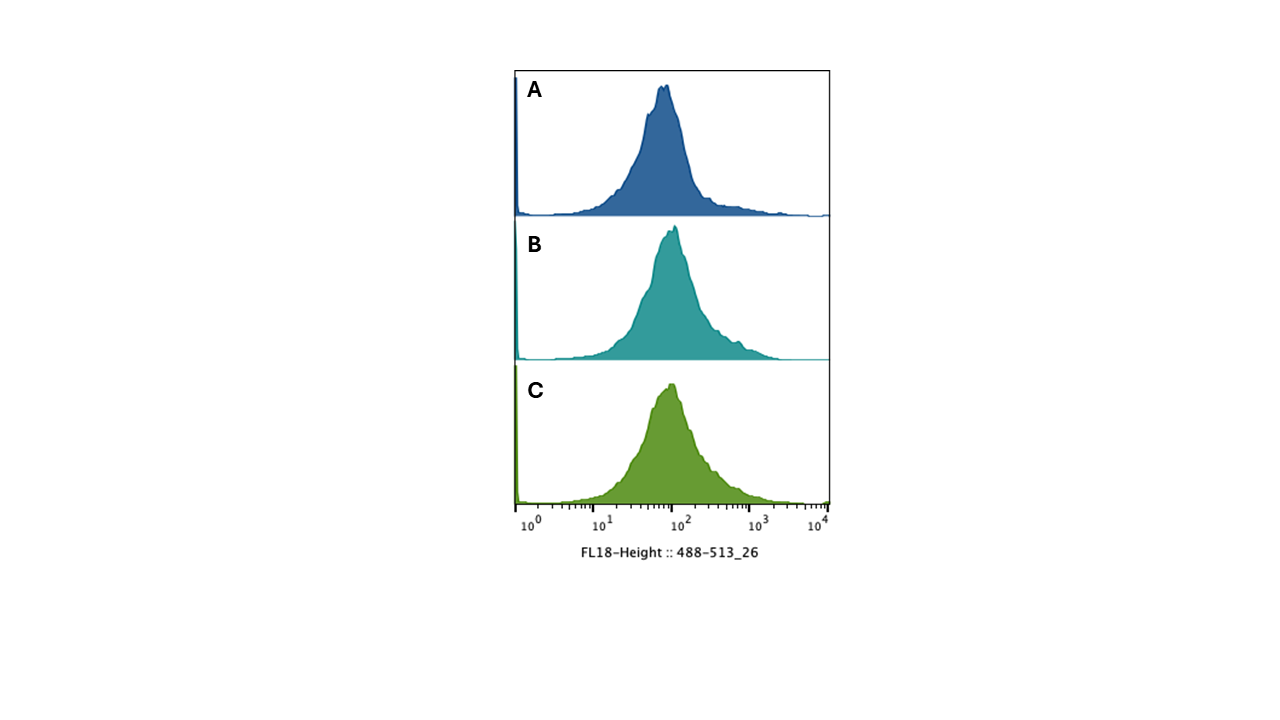


Figure S6.

Flow cytometry analysis of the HeLa cells expressing EGFP without the US-responsive RNA riboswitch. (**A**) without any treatment and (**B**) after treatment with 1 mM pristine NeoB and 1 MHz FUS for 30 min. (**C**) Shows cells expressing EGFP and the RNA riboswitch.


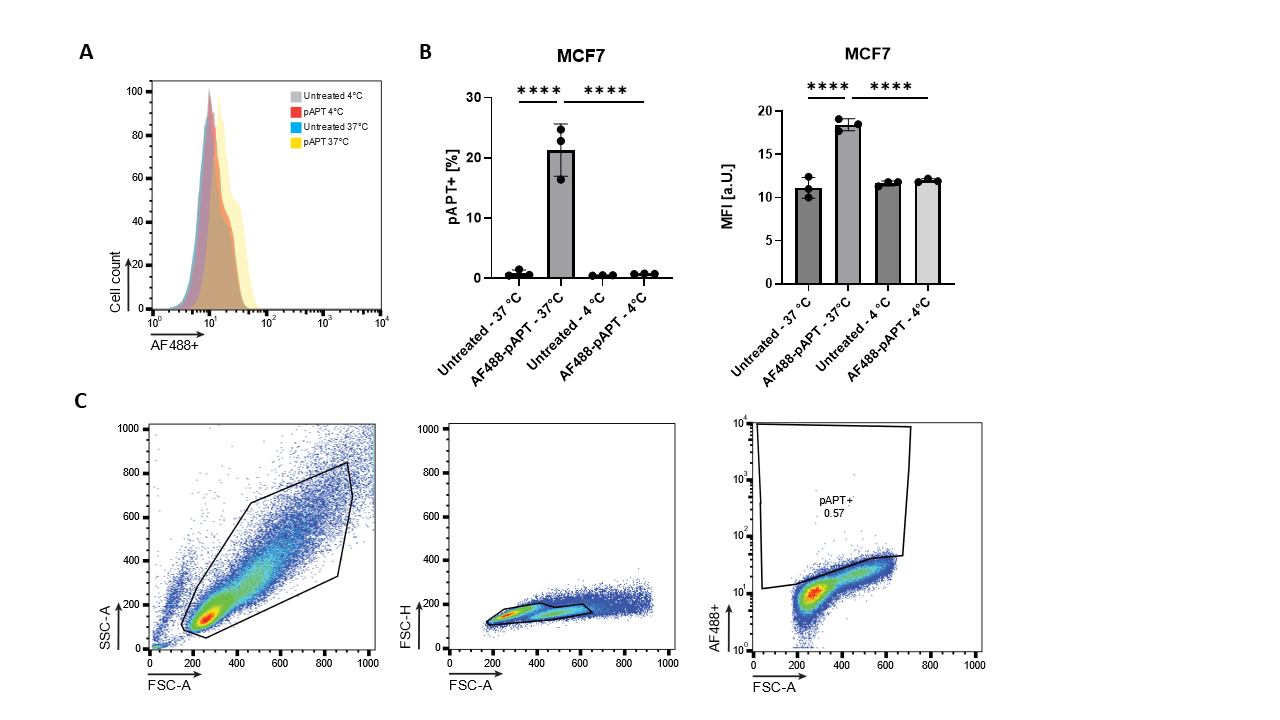


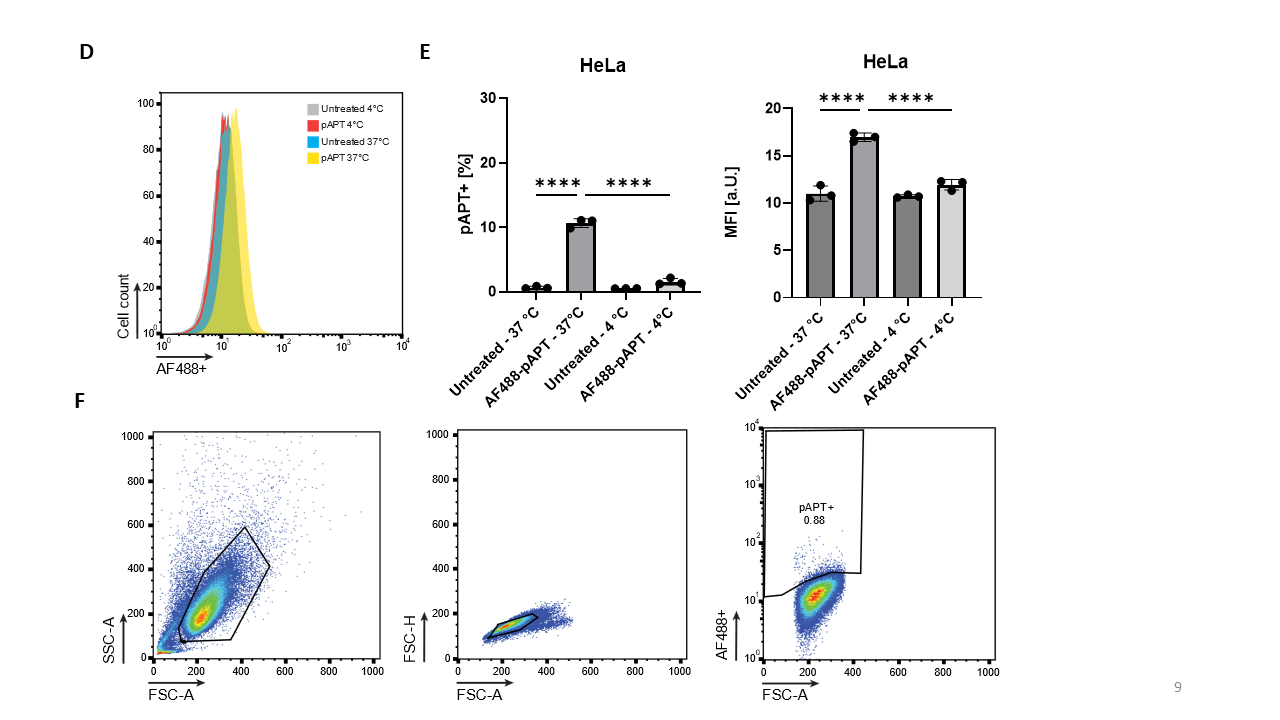


Figure S7.

Cellular uptake of fluorescently labelled pAPT in mammalian cells, tracked by flow cytometry in MCF7 (**A**) and HeLa (**D**) cells. Quantitative analysis of pAPT uptake after incubation at 37 °C and 4 °C for 4 h with either MCF7 (**B**) or HeLa (**E**) cells. Gating strategy used for cellular uptake experiments for MCF7 (**C**) or HeLa (**F**) cells showing gating for live cells (left), singlets (middle) and AF488+ population (right).

Table S1.

Sequences of DNA and RNA oligonucleotides used for RCT reaction (5’ to 3’).

| **R23 Aptamer** | GGACUGGGCGAGAAGUUUAGUCC |
| --- | --- |
| **T7 Promoter** | TAATACGACTCACTATAGGGAT |
| **RCT Template** | Phosphate-ATAGTGAGTCGTATTATTTTGGCCTAAACTTCTC GCCCAGGCCTTTTTTTTTTTTTTATCCCT |

Table S2.

Sequences of aptamer and plasmids (5’ to 3’).

| **N1 Aptamer** | GACTGCTTGTCCTTTAATGGTCCAGTC |
| --- | --- |
| N1-pWHE601 sequence including:  ADH1 promoter**-**N1 aptamer**-** GFP**-** ADH1 terminator**-** 2M ori**-** URA3**-** URA3 promoter | CGACCTCGACGCATGCAACTTCTTTTCTTTTTTTTTCTTTTCTCTCTCCCCCGTTGTTGTCTCACCATATCCGCAATGACAAAAAAAATGATGGAAGACACTAAAGGAAAAAATTAACGACAAAGACAGCACCAACAGATGTCGTTGTTCCAGAGCTGATGAGGGGTATCTCGAAGCACACGAAACTTTTTCCTTCCTTCATTCACGCACACTACTCTCTAATGAGCAACGGTATACGGCCTTCCTTCCAGTTACTTGAATTTGAAATAAAAAAAAGTTTGCTGTCTTGCTATCAAGTATAAATAGACCTGCAATTATTAATCTTTTGTTTCCTCGTCATTGTTCTCGTTCCCTTTCTTCCTTGTTTCTTTTTCTGCACAATATTTCAAGCTATACCAAGCATACAATCAACTCCAAGCTAGATCTCTTAAGGACTGCTTGTCCTTTAATGGTCCAGTCAAAACGATGGCTAGCAAAGGAGAAGAACTTTTCACTGGAGTTGTCCCAATTCTTGTTGAATTAGATGGTGATGTTAATGGGCACAAATTTTCTGTCAGTGGAGAGGGTGAAGGTGATGCTACATACGGAAAGCTTACCCTTAAATTTATTTGCACTACTGGAAAACTACCTGTTCCATGGCCAACACTTGTCACTACTTTGACCTATGGTGTTCAATGCTTTTCCCGTTATCCGGATCATATGAAACGGCATGACTTTTTCAAGAGTGCCATGCCCGAAGGTTATGTACAGGAACGCACTATATCTTTCAAAGATGACGGGAACTACAAGACGCGTGCTGAAGTCAAGTTTGAAGGTGATACCCTTGTTAATCGTATCGAGTTAAAAGGTATTGATTTTAAAGAAGATGGAAACATTCTCGGACACAAACTCGAGTACAACTATAACTCACACAATGTATACATCACGGCAGACAAACAAAAGAATGGAATCAAAGCTAACTTCAAAATTCGCCACAACATTGAAGATGGATCCGTTCAACTAGCAGACCATTATCAACAAAATACTCCAATTGGCGATGGCCCTGTCCTTTTACCAGACAACCATTACCTGTCGACACAATCTGCCCTTTCGAAAGATCCCAACGAAAAGCGTGACCACATGGTCCTTCTTGAGTTTGTAACTGCTGCTGGGATTACACATGGCATGGATGAGCTCTACAAATAATGAATTCGAGCATTTAAATCTAGACGAGCTCCTCGAGCCTGCAGCAGCTGAAGCTTTGGACTTCTTCGCCAGAGGTTTGGTCAAGTCTCCAATCAAGGTTGTCGGCTTGTCTACCTTGCCAGAAATTTACGAAAAGATGGAAAAGGGTCAAATCGTTGGTAGATACGTTGTTGACACTTCTAAATAAGCGAATTTCTTATGATTTATGATTTTTATTATTAAATAAGTTATAAAAAAAATAAGTGTATACAAATTTTAAAGTGACTCTTAGGTTTTAAAACGAAAATTCTTATTCTTGAGTAACTCTTTCCTGTAGGTCAGGTTGCTTTCTCAGGTATAGCATGAGGTCGCTCTTATTGACCACACCTCTACCGGCATGCGATATGATCCAATATCAAAGGAAATGATAGCATTGAAGGATGAGACTAATCCAATTGAGGAGTGGCAGCATATAGAACAGCTAAAGGGTAGTGCTGAAGGAAGCATACGATACCCCGCATGGAATGGGATAATATCACAGGAGGTACTAGACTACCTTTCATCCTACATAAATAGACGCATATAAGTACGCATTTAAGCATAAACACGCACTATGCCGTTCTTCTCATGTATATATATATACAGGCAACACGCAGATATAGGTGCGACGTGAACAGTGAGCTGTATGTGCGCAGCTCGCGTTGCATTTTCGGAAGCGCTCGTTTTCGGAAACGCTTTGAAGTTCCTATTCCGAAGTTCCTATTCTCTAGCTAGAAAGTATAGGAACTTCAGAGCGCTTTTGAAAACCAAAAGCGCTCTGAAGACGCACTTTCAAAAAACCAAAAACGCACCGGACTGTAACGAGCTACTAAAATATTGCGAATACCGCTTCCACAAACATTGCTCAAAAGTATCTCTTTGCTATATATCTCTGTGCTATATCCCTATATAACCTACCCATCCACCTTTCGCTCCTTGAACTTGCATCTAAACTCGACCTCTACATTTTTTATGTTTATCTCTAGTATTACTCTTTAGACAAAAAAATTGTAGTAAGAACTATTCATAGAGTGAATCGAAAACAATACGAAAATGTAAACATTTCCTATACGTAGTATATAGAGACAAAATAGAAGAAACCGTTCATAATTTTCTGACCAATGAAGAATCATCAACGCTATCACTTTCTGTTCACAAAGTATGCGCAATCCACATCGGTATAGAATATAATCGGGGATGCCTTTATCTTGAAAAAATGCACCCGCAGCTTCGCTAGTAATCAGTAAACGCGGGAAGTGGAGTCAGGCTTTTTTTATGGAAGAGAAAATAGACACCAAAGTAGCCTTCTTCTAACCTTAACGGACCTACAGTGCAAAAAGTTATCAAGAGACTGCATTATAGAGCGCACAAAGGAGAAAAAAAGTAATCTAAGATGCTTTGTTAGAAAAATAGCGCTCTCGGGATGCATTTTTGTAGAACAAAAAAGAAGTATAGATTCTTTGTTGGTAAAATAGCGCTCTCGCGTTGCATTTCTGTTCTGTAAAAATGCAGCTCAGATTCTTTGTTTGAAAAATTAGCGCTCTCGCGTTGCATTTTTGTTTTACAAAAATGAAGCACAGATTCTTCGTTGGTAAAATAGCGCTTTCGCGTTGCATTTCTGTTCTGTAAAAATGCAGCTCAGATTCTTTGTTTGAAAAATTAGCGCTCTCGCGTTGCATTTTTGTTCTACAAAATGAAGCACAGATGCTTCGTTAACAAAGATATGCTATTGAAGTGCAAGATGGAAACGCAGAAAATGAACCGGGGATGCGACGTGCAAGATTACCTATGCAATAGATGCAATAGTTTCTCCAGGAACCGAAATACATACATTGTCTTCCGTAAAGCGCTAGACGTATATATTATTATACAGGTTCAAATATACTATCTGTTTCAGGGAAAACTCCCAGGTTCGGAGTTCAAAATTCAATGATGGGTACAAGTACGATCGTAAATCTGTAAAACAGTTTGTCGGATATTAGGCTGTATCTCCTCAAAGCGTATTCGAATATCATTGAGGAGCTGCATTTTTTTTTTTTTTTTTTTTTTTTTTTTTTATATATATTTCAAGGATATACCATTGTAATGTCTGCCCCTAAGAAGATCGTCGTTTTGCCAGGTGACCACGTTGGTCAAGAAATCACAGCCGAAGCCATTAAGGTTCTTAAAGCTATTTCNGATGTTCGTTCCAATGTCAAGTTCGATTTCGAAAATCATTTAATTGGTGGTGCTGCTATCGCAGATCCAGATCTGGCTTTTTCTTTCCAATTTTTTTTTTTTCGTCATTATAAAAATCATTACGACCGAGATTCCCGGGTAATAACTGATATAATTAAATTGAAGCTCTAATTTGTGAGTTTAGTATACATGCATTTACTTATAATACAGTTTTTTAGTTTTGCTGGCCGCATCTTCTCAAATATGCTTCCCAGCCTGCTTTTCTGTAACGTTCACCCTCTACCTTAGCATCCCTTCCCTTTGCAAATAGTCCTCTTCCAACAATAATAATGTCAGATCCTGTAGAGACCACATCATCCACGGTTCTATACTGTTGACCCAATGCGTCTCCCTTGTCATCTAAACCCACACCGGGTGTCATAATCAACCAATCGTAACCTTCATCTCTTCCACCCATGTCTCTTTGAGCAATAAAGCCGATAACAAAATCTTTGTCGCTCTTCGCAATGTCAACAGTACCCTTAGTATATTCTCCAGTAGATAGGGAGCCCTTGCATGACAATTCTGCTAACATCAAAAGGCCTCTAGGTTCCTTTGTTACTTCTTCTGCCGCCTGCTTCAAACCGCTAACAATACCTGGGCCCACCACACCGTGTGCATTCGTAATGTCTGCCCATTCTGCTATTCTGTATACACCCGCAGAGTACTGCAATTTGACTGTATTACCAATGTCAGCAAATTTTCTGTCTTCGAAGAGTAAAAAATTGTACTTGGCGGATAATGCCTTTAGCGGCTTAACTGTGCCCTCCATGGAAAAATCAGTCAAGATATCCACATGTGTTTTTAGTAAACAAATTTTGGGACCTAATGCTTCAACTAACTCCAGTAATTCCTTGGTGGTACGAACATCCAATGAAGCACACAAGTTTGTTTGCTTTTCGTGCATGATATTAAATAGCTTGGCAGCAACAGGACTAGGATGAGTAGCAGCACGTTCCTTATATGTAGCTTTCGACATGATTTATCTTCGTTTCCGGTTTTTGTTCTGTGCAGTTGGGTTAAGAATACTGGGCAATTTCATGTTTCTTCAACACTACATATGCGTATATATACCAATCTAAGTCTGTGCTCCTTCCTTCGTTCTTCCTTCTGTTCGGAGATTACCGAATCAAAAAAATTTCAAGGAAACCGAAATCAAAAAAAAGAATAAAAAAAAAATGATGAATTGAA |
| **N4-HHR Aptazyme** | CAAAGCGCGTCCTGCTCTGCTTGTCCTTTAATGGTCCAGTCCTATCTATCCACGGTACTAGCAGCTGATGAGTCCCAAATAGGACGAAACGCGCAAAA |
| Halo-EGFP-N4-HHR  CMV enhancer**-** CMV promoter-T7 promoter**-**EGFP**-N4-HHR9-**bGH poly(A) signal | CAATTGCATGAAGAATCTGCTTAGGGTTAGGCGTTTTGCGCTGCTTCGCGATGTACGGGCCAGATATACGCGTTGACATTGATTATTGACTAGTTATTAATAGTAATCAATTACGGGGTCATTAGTTCATAGCCCATATATGGAGTTCCGCGTTACATAACTTACGGTAAATGGCCCGCCTGGCTGACCGCCCAACGACCCCCGCCCATTGACGTCAATAATGACGTATGTTCCCATAGTAACGCCAATAGGGACTTTCCATTGACGTCAATGGGTGGAGTATTTACGGTAAACTGCCCACTTGGCAGTACATCAAGTGTATCATATGCCAAGTACGCCCCCTATTGACGTCAATGACGGTAAATGGCCCGCCTGGCATTATGCCCAGTACATGACCTTATGGGACTTTCCTACTTGGCAGTACATCTACGTATTAGTCATCGCTATTACCATGGTGATGCGGTTTTGGCAGTACATCAATGGGCGTGGATAGCGGTTTGACTCACGGGGATTTCCAAGTCTCCACCCCATTGACGTCAATGGGAGTTTGTTTTGGCACCAAAATCAACGGGACTTTCCAAAATGTCGTAACAACTCCGCCCCATTGACGCAAATGGGCGGTAGGCGTGTACGGTGGGAGGTCTATATAAGCAGAGCTCTCTGGCTAACTAGAGAACCCACTGCTTACTGGCTTATCGAAATTAATACGACTCACTATAGGGAGACCCAAGCTGGCTAGCGTTTAAACTTAAGCTTATGTACCCTTATGACGTACCTGACTATGCTGGAGTGAGCAAGGGCGAGGAGCTGTTCACCGGGGTGGTGCCCATCCTGGTCGAGCTGGACGGCGACGTAAACGGCCACAAGTTCAGCGTGTCCGGCGAGGGCGAGGGCGATGCCACCTACGGCAAGCTGACCCTGAAGTTCATCTGCACCACCGGCAAGCTGCCCGTGCCCTGGCCCACCCTCGTGACCACCCTGACCTACGGCGTGCAGTGCTTCAGCCGCTACCCCGACCACATGAAGCAGCACGACTTCTTCAAGTCCGCCATGCCCGAAGGCTACGTCCAGGAGCGCACCATCTTCTTCAAGGACGACGGCAACTACAAGACCCGCGCCGAGGTGAAGTTCGAGGGCGACACCCTGGTGAACCGCATCGAGCTGAAGGGCATCGACTTCAAGGAGGACGGCAACATCCTGGGGCACAAGCTGGAGTACAACTACAACAGCCACAACGTCTATATCATGGCCGACAAGCAGAAGAACGGCATCAAGGTGAACTTCAAGATCCGCCACAACATCGAGGACGGCAGCGTGCAGCTCGCCGACCACTACCAGCAGAACACCCCCATCGGCGACGGCCCCGTGCTGCTGCCCGACAACCACTACCTGAGCACCCAGTCCGCCCTGAGCAAAGACCCCAACGAGAAGCGCGATCACATGGTCCTGCTGGAGTTCGTGACCGCCGCCGGGATCACTCTCGGCATGGACGAGCTGTACAAGGCTCGAGATACAAACTCATCCGAAATCGGTACAGGCTTCCCCTTCGACCCCCATTATGTGGAAGTCCTGGGCGAGCGTATGCACTACGTCGATGTTGGACCGCGGGATGGCACGCCTGTGCTGTTCCTGCACGGTAACCCGACCTCGTCCTACCTGTGGCGCAACATCATCCCGCATGTAGCACCGAGTCATCGGTGCATTGCTCCAGACCTGATCGGGATGGGAAAATCGGACAAACCAGACCTCGATTATTTCTTCGACGACCACGTCCGCTACCTCGATGCCTTCATCGAAGCCTTGGGTTTGGAAGAGGTCGTCCTGGTCATCCACGACTGGGGCTCAGCTCTCGGATTCCACTGGGCCAAGCGCAATCCGGAACGGGTCAAAGGTATTGCATGTATGGAATTCATCCGGCCTATCCCGACGTGGGACGAATGGCCAGAATTCGCCCGTGAGACCTTCCAGGCCTTCCGGACCGCCGACGTCGGCCGAGAGTTGATCATCGATCAGAACGCTTTCATCGAGGGTGCGCTCCCGATGGGGGTCGTCCGTCCGCTTACGGAGGTCGAGATGGACCACTATCGCGAGCCCTTCCTCAAGCCTGTTGACCGAGAGCCACTGTGGCGATTCCCCAACGAGCTGCCCATCGCCGGTGAGCCCGCGAACATCGTCGCGCTCGTCGAGGCATACATGAACTGGCTGCACCAGTCACCTGTCCCGAAGTTGTTGTTCTGGGGCACACCCGGCGTACTGATCCCCCCGGCCGAAGCCGCGAGACTTGCCGAAAGCCTCCCCAACTGCAAGACAGTGGACATCGGCCCGGGATTGTTCTTGCTCCAGGAAGACAACCCGGACCTTATCGGCAGTGAGATCGCGCGCTGGCTCCCCGGGCTGGCCGGCTAAGATATCCAGCACAGTGCAAAGCGCGTCCTGCTCTGCTTGTCCTTTAATGGTCCAGTCCTATCTATCCACGGTACTAGCAGCTGATGAGTCCCAAATAGGACGAAACGCGCAAAAGCGGCCGCTCGAGTCTAGAGGGCCCGTTTAAACCCGCTGATCAGCCTCGACTGTGCCTTCTAGTTGCCAGCCATCTGTTGTTTGCCCCTCCCCCGTGCCTTCCTTGACCCTGGAAGGTGCCACTCCCACTGTCCTTTCCTAATAAAATGAGGAAATTGCATCGCATTGTCTGAGTAGGTGTCATTCTATTCTGGGGGGTGGGGTGGGGCAGGACAGCAAGGGGGAGGATTGGGAAGACAATAGCAGGCATGCTGGGGATGCGGTGGGCTCTATGGCTTCT |

Table S3.

Sequences of primers (5’ to 3’).

| **Seq-pWH FW** | CGGCCTTCCTTCCAGTTACTTG |
| --- | --- |
| **Seq-pWH RV** | GGCGAAGAAGTCCAAAGCTTCAG |
| **5Cap-Neo+2 FW** | GTACATCAATGGGCGTGGATAG |
| **Neo-HHR RV** | CAGCATGCCTGCTATTGTCTTC |
